# Anti-Myeloma Activity of Akt Inhibition Is Linked to the Activation Status of PI3K/Akt and MEK/ERK Pathway

**DOI:** 10.1371/journal.pone.0050005

**Published:** 2012-11-21

**Authors:** Vijay Ramakrishnan, Teresa Kimlinger, Jessica Haug, Utkarsh Painuly, Linda Wellik, Timothy Halling, S. Vincent Rajkumar, Shaji Kumar

**Affiliations:** The Divisions of Hematology, Mayo Clinic, Rochester, Minnesota, United States of America; The University of Kansas Medical Center, United States of America

## Abstract

The PI3K/Akt/mTOR signal transduction pathway plays a central role in multiple myeloma (MM) disease progression and development of therapeutic resistance. mTORC1 inhibitors have shown limited efficacy in the clinic, largely attributed to the reactivation of Akt due to rapamycin induced mTORC2 activity. Here, we present promising anti-myeloma activity of MK-2206, a novel allosteric pan-Akt inhibitor, in MM cell lines and patient cells. MK-2206 was able to induce cytotoxicity and inhibit proliferation in all MM cell lines tested, albeit with significant heterogeneity that was highly dependent on basal pAkt levels. MK-2206 was able to inhibit proliferation of MM cells even when cultured with marrow stromal cells or tumor promoting cytokines. The induction of cytotoxicity was due to apoptosis, which at least partially was mediated by caspases. MK-2206 inhibited pAkt and its down-stream targets and up-regulated pErk in MM cells. Using MK-2206 in combination with rapamycin (mTORC1 inhibitor), LY294002 (PI3K inhibitor), or U0126 (MEK1/2 inhibitor), we show that Erk- mediated downstream activation of PI3K/Akt pathway results in resistance to Akt inhibition. These provide the basis for clinical evaluation of MK-2206 alone or in combination in MM and potential use of baseline pAkt and pErk as biomarkers for patient selection.

## Introduction

Multiple myeloma (MM) is an incurable plasma cell neoplasm that has seen considerable improvement in patient survival in the last decade due to the introduction of several effective therapies [1]. However, the responders eventually relapse and become refractory to current therapies [1]. Novel drugs based on better understanding of the disease biology are urgently required to overcome resistance and improve patient outcomes in MM [2]. Phosphotidylinositol-3-kinase (PI3K) represents a family of serine threonine kinases that initiate a complex signaling cascade in response to the binding of extracellular cytokines to their receptors expressed on cellular membranes [3]. When activated, the PI3Ks phosphorylate phosphotidylinositol-4,5-bisphosphate (PIP_2_) to PIP_3_. PIP_3_ binds to the PH domain of Akt, which causes a conformational change in Akt exposing amino acids (Thr308 and Ser473) that are then phosphorylated and activated [4], [5]. Akt then phosphorylates and modulates multiple proteins leading to increased cell growth and survival, decreased apoptosis and drug resistance [6], [7], [8], [9], [10], [11], [12], [13]. A critical down-stream member of the PI3K/Akt pathway is the mammalian target of rapamycin complex I (mTORC1) which is activated by Akt either directly by relieving the PRAS40 mediated inhibition of mTORC1 or indirectly through the inhibition of TSC2 [14], [15]. Once activated, mTORC1 regulates cell growth, metabolism, translation and autophagy [16], [17].

In MM, activating mutations of PI3K/Akt/mTOR pathway members or inactivating mutations of the tumor suppressor PTEN are uncommon events [18], [19]. However, the pathway is up regulated in a significant proportion of MM patients due to the interaction of MM cells with non-malignant cells in the microenvironment, increased levels of tumor promoting cytokines and activating mutations or aberrant expression levels of other signaling pathways that feed into the PI3K/Akt pathway [20], [21], [22], [23], [24]. Activated PI3K/Akt pathway inactivates caspase-9 and offers resistance against Dexamethasone induced apoptosis [20]. IL6 stimulated PI3K/Akt signaling has also been found to phosphorylate and inactivate forkhead transcriptional factor (FKHR) resulting in G1/S phase transition, whereas PI3K inhibitors such as LY294002 block this signaling, resulting in up regulation of p27 (KIP1) and G1 growth arrest [20]. Prevention of IL6 induced mTOR activity by rapamycin and CCI779 results in inhibition of proliferation [23]. IGF1 stimulation also leads to activation of the PI3K/Akt pathway, phosphorylation of the FKHRL1 Forkhead transcription factor and up regulation of the anti apoptotic proteins FLIP, survivin, cIAP2, A1/Bfl1, and Xiap [22].

Thus, it is clear that this pathway is critical to MM pathogenesis and resistance to therapy. However, inhibiting this pathway at the mTOR kinase level has seen limited success in the clinic [25]. Increased pAKT (Ser 473) levels post rapamycin or rapalog treatment has been attributed to be an important factor in the resistance to these drugs [26]. In addition, down regulation of activated p70S6K, a substrate of mTOR, by mTOR inhibitors also leads to the activation of the pathway upstream of PI3K [27]. We therefore examined the effect of MK-2206, a small molecule Akt kinase specific allosteric inhibitor, in both MM cell lines and patient cells.

## Materials and Methods

### Ethics

Bone marrow aspirates were collected from MM patients after written informed consent. The Mayo Clinic Institutional Review Board approved the study in adherence with the Declaration of Helsinki.

### Multiple Myeloma Cell Lines, Bone Marrow Stromal Cells and Patient Cells

Dexamethasone sensitive (MM1.S) and resistant (MM1.R) human MM cell lines both from Dr. Steven Rosen’s laboratory (Northwestern University, Chicago, IL) [28]; doxorubicin resistant (DOX 40), and melphalan resistant (LR5) RPMI 8226 human MM cell lines were from Dr. William Dalton’s laboratory (Moffitt Cancer Center, Tampa, FL) [29], [30] and sensitive RPMI 8226 cell line, NCI-H929 and U266 cell lines were purchased from American Type Culture Collection (ATCC) (Manassas, VA). OPM2 was obtained from Dr. Leif Bergsagel’s laboratory (Mayo Clinic, Scottsdale, AZ) [31]. All cell lines were cultured in RPMI 1640 media (Mediatech Inc., Manassas, VA) containing 10% fetal bovine serum (Mediatech, Inc.), 2 mM L-glutamine (Invitrogen, Grand Island, NY), 100 U/mL penicillin, and 100 µg/mL streptomycin (Invitrogen). Freshly obtained bone marrow aspirates from patients were collected with informed consent and were processed to obtain myeloma cells or stromal cells as previously described [32], [33], [34]. All patient cells were cultured in RPMI 1640 media that contained 10% fetal bovine serum, 2 mM L-glutamine (GIBCO), 100 U/mL penicillin, and 100 µg/mL streptomycin.

### Reagents

MK-2206 was provided by Merck. & Co., Inc. (Whitehouse Station, NJ) under a Material Transfer Agreement. Rapamycin (Sirolimus) and dexamethasone were purchased from EMD Chemicals Inc. (Gibbstown, NJ). LY294002 and U0126 were purchased from cell signaling (Danvers, MA). Stock solutions (10 mM) of each of the above drugs except rapamycin were made in DMSO, aliquoted and stored at −20°C. Rapamycin stock solution (5 mM) was made in DMSO. Pan-caspase inhibitor Q-VD-OPH, caspase 9 inhibitor Ac-LEHD-CMK and caspase 8 inhibitor Z-IETD-FMK were purchased from EMD Chemicals Inc.

### MTT and Proliferation Assays

Viability of MM cells post MK-2206 treatment was measured using 3-(4, 5-dimethylthiazol-2-yl)-2, 5-diphenyl tetrasodium bromide (MTT) (Chemicon International Inc., Temecula, CA) colorimetric assay and proliferation of MM cells post MK-2206 treatment was measured by tritiated thymidine uptake as previously described [32], [33], [34]. Experiments were performed in triplicate.

### Apoptosis Assay

Apoptosis of MM cell lines was assayed as described before [32], [33], [34]. Briefly, control or treated cells were washed twice with annexin binding buffer (ABB) (10 mM HEPES pH 7.4, 140 mM NaCl, 2.5 mM CaCl_2_). 100 µl cells (10^7^ cells per ml) were stained with 3 µl of annexin V- FITC (Caltag, Burlingame, CA) for 15 mins at room temperature. Cells were washed again with ABB and resuspended in 500 µl of ABB containing 5 µl of 1 mg/ml propidium iodide (PI) (Sigma-Aldrich, St. Louis, MO). The samples were subsequently run on a Canto flow cytometer (BD Biosciences, San Jose, CA). Experiments were performed in triplicate.

### Caspase Assay

Activated levels of caspases 3, 8 and 9 were measured indirectly using kits from OncoImmunin (Gaithersburg, MD). PhiPhiLux G1D2 (catalog# A304R1G-3) was used for the detection of caspase 3, CaspaLux 8 L1D2 (catalog# CPL8R1L-3) for caspase 8 and CaspaLux 9 M1D2 (catalog# CPL9R1M-3) was used for the detection of caspase 9. Samples were run on the Canto flow cytometer (BD Biosciences). Experiments were performed in triplicate.

### Western Blotting

MM cell lines or patient cells were incubated with indicated concentrations of MK-2206 for indicated time periods. Cells were harvested and lysed with RIPA buffer (50 mM HEPES (pH 7.4), 150 mM NaCl, 1% Triton X-100, 30 mM sodium pyrophosphate, 5 mM EDTA, 2 mM Na_3_VO_4_, 5 mM NaF, 1 mM phenylmethyl-sulfonyl-fluoride (PMSF) and protease inhibitor cocktail). Protein lysate concentrations were measured using BCA assay (Pierce, Rockford, IL). Equal amounts of protein were loaded on 12% Tris-Glycine gels and transferred onto nitrocellulose membranes. Antibodies used pAkt (Ser473), pAkt (Thr308), Akt, pErk, Erk, pmTOR, mTOR, p-p70S6K, p70S6K, p4EBP1, 4EBP1, pS6, S6, pGsk3β, Gsk3β, pFoxO3A, FoxO3A, pStat3, Stat3, pBad, Bad, Bcl2, Bcl-Xl, Mcl1, Xiap and β actin were all purchased from Cell Signaling. Antigen-antibody complexes were detected using enhanced chemiluminescence (Amersham, Arlington Heights, IL). Experiments on MM cell lines were performed on three separate occasions.

### Isobologram Analysis

The effects of MK-2206 in combination with rapamycin, LY294002, U0126 or dexamethasone on MM cells were analyzed using the CalcuSyn™ software program (Biosoft, Ferguson, MO). This program is based upon the Chou-Talalay method, which calculates a combination index (CI), and analysis is performed based on the following equation: CI = (D)1/(Dx)1+(D)2/(Dx)2+(D)1(D)2/(Dx)1(Dx)2, where (D)1 and (D)2 are the doses of drug 1 and drug 2 that have×effect when used in combination, and (Dx)1 and (Dx)2 are the doses of drug 1 and drug 2 that have the same×effect when used alone [35]. Data from the MTT viability assay was expressed as the fraction of cells killed by the individual drug or the combination in drug-treated cells compared with untreated cells. A CI of 1.0 indicates an additive effect, whereas CI values below 1.0 indicate synergism.

## Results

### MK-2206 Induces Cytotoxicity and Inhibits Proliferation in MM Cell Lines

**Figure 1:**
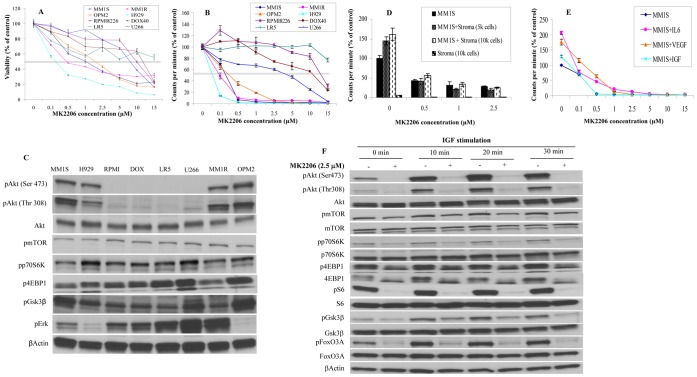
MK-2206 induced cytotoxicity, inhibited proliferation and was able to overcome the protective effects of the tumor microenvironment. When we cultured MM cell lines with indicated concentrations of MK-2206 for 48 hrs we observed dose dependent decrease in **A)** viability as observed by MTT assays and **B)** tritiated thymidine uptake indicative of inhibition of proliferation albeit with differences in the sensitivity of cell lines to MK-2206. MK-2206 concentration is indicated along the×axis and in **A)** viability is expressed as % of control is indicated along the Y axis and in B) proliferation is expressed as % cpm of control. Error bars represent standard deviation. **C)** We evaluated baseline levels of activated Akt and down-stream members of the PI3K/Akt/mTOR pathway including pmTOR, p-p70S6K, p4EBP1 and in addition pErk levels by western blotting. We observed that cell lines sensitive to MK-2206 expressed high levels of pAkt whereas the cell lines less sensitive to MK-2206 expressed low levels of pAkt but high levels of pErk. When MM1S cells were co-cultured with **D)** bone marrow stromal cells (BMSCs) or **E)** cytokines IL6 (25 ng/ml), IGF (50 ng/ml) or VEGF (50 ng/ml), we observed increase in proliferation measured by increase in cpm. MK-2206 was able to inhibit this increase in proliferation. MK-2206 concentrations are indicated on X-axis and % cpm is indicated on the Y-axis. Error bars represent standard deviation. **F)** We cultured MM1S cells with or without 2.5 µM of MK-2206 for 8 hrs. In the last thirty minutes of incubation, cells were treated with IGF (50 ng/ml) for indicated time points. We then made lysates and checked for the ability of MK-2206 to inhibit pAkt and down-stream members of the PI3K/Akt/mTOR pathway. We observed potent down regulation of pAkt and downstream members with no differences observed in total proteins and actin, which serve as loading controls.

Incubating MM cells with indicated concentrations of MK-2206 for 48 hrs showed heterogeneity among MM cell lines in their sensitivity to the drug. MM1S, MM1R, OPM2 and H929 cells were sensitive to drug treatment with IC50 values in the range of 0.5–2.5 µM **(** **Figure 1A** **and Table S1)**. However, RPMI, DOX40 and U266 cells were sensitive only at higher concentrations of MK-2206 with IC50 values in the range of 10–15 µM **(** **Figure 1A** **and Table S1)**. IC50 was not reached in LR5 cells. Similar differences were observed when the cells were treated with MK-2206 and proliferation was studied **(** **Figure 1B** **)**. We then examined if there is a correlation between basal levels of activated Akt pathway members and sensitivity to MK-2206. Like in a previous study [36], we observed constitutively activated Akt (both Ser 473 and Thr 308) in MM1S, MM1R, OPM2 and H929 cells **(** **Figure 1C** **)**. We did not observe activated Akt in RPMI8226, DOX40, LR5 and U266 cells, which were resistant to MK-2206 treatment **(** **Figure 1C** **)**. However, we observed constitutively activated mTOR and downstream substrates of mTOR including p70S6K and 4EBP1 in these cell lines **(** **Figure 1C** **)**. This suggested that in these cell lines, the downstream pathway is independent of Akt status. It is known that oncogenic Ras could activate mTOR in MM [21]. While RPMI8226, D0X40 and LR5 all have K-Ras mutations, U266 has wild type K-Ras and hence Ras mutation status alone cannot predict for activation of mTOR independent of Akt. It is also known that the MEK/Erk pathway can be activated in MM irrespective of Ras mutation [34]. Consistent with this possibility, we observed high levels of activated Erk in U266, RPMI8226, DOX40, and LR5, suggesting a non-Akt dependent up-regulation of the downstream mediators explaining the resistance seen with Akt inhibition in these cell lines **(** **Figure 1C** **)**. Thus, our results indicate that constitutively activated Akt predicts for sensitivity to MK-2206.

### MK-2206 Overcomes the Protective Effect of Bone Marrow Microenvironment

The components of the microenvironment including the bone marrow stromal cells (BMSCs) and cytokines such as IL6, IGF and VEGF are well known to be important mediators of tumor growth and drug resistance in MM. We therefore wanted to examine if MK-2206 was able to overcome the tumor protective effects observed when MM cells were co-cultured with BMSCs or these growth factors. As shown in **Figures 1D–E**, MK-2206 overcame the stimulatory effect of the microenvironment and inhibited the proliferation of MM1S cells when co-cultured with BMSCs or cytokines (IL6, IGF or VEGF) at concentration similar to that seen with MM cells alone. Given the importance of IGF-1 in myeloma biology, we specifically examined the effect of MK-2206 on inhibiting PI3K/Akt/mTOR pathway in the presence of IGF. MK-2206 was clearly able to prevent the activation of pAkt and the canonical pathway members, which were otherwise activated by IGF **(** **Figure 1F** **)**.

### MK-2206 Induces Apoptosis in MM Cell Lines and Patient Myeloma Cells

**Figure 2:**
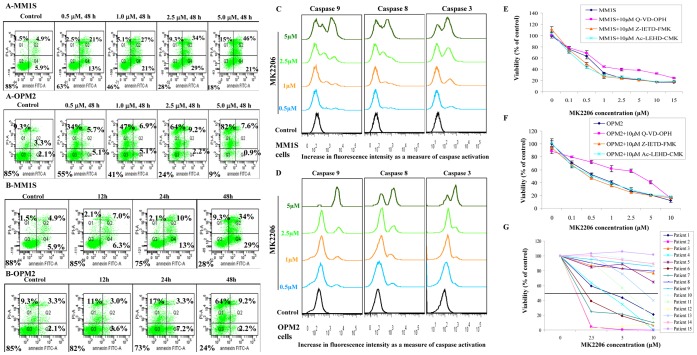
MK-2206 induced apoptosis in MM cell lines and patient cells. We incubated MM1S and OPM2 cells with **A)** indicated concentrations of MK-2206 for 48 hrs and **B)** with 2.5 µM of MK-2206 for 12, 24 or 48 hrs. We observed dose and time dependent increase in apoptosis as measured by annexin/PI staining. % cells in each quadrant is indicated. **C)** MM1S or **D)** OPM2 with indicated concentrations of MK-2206 for 48 hrs and measured activation of caspases 9, 8 and 3. We observed dose dependent increase in activation of each of the three caspases. In all the above experiments, control refers to cells untreated with MK-2206. We incubated **E)** MM1S or **F)** OPM2 cells with indicated concentrations of MK-2206, MK-2206 plus Q-VD-OPH (10 µM), MK-2206 plus Z-IETD-FMK (10 µM) or MK-2206 plus Ac-LEHD-CMK(10 µM) for 48 hrs. We observed dose dependent decrease in cell viability, which was partially blocked by Q-VD-OPH. MK-2206 concentration is indicated along×axis and viability (% of control) is indicated along Y-axis. Error bars represent standard deviation. **G)** We incubated cells from MM patients with indicated concentrations of MK-2206 for 48 hrs. We then measured for plasma cells undergoing apoptosis by annexin/PI staining. MK-2206 concentration is indicated along×axis and viability (% of control) is indicated along Y axis.

We then wanted to examine if the cytotoxic effects observed were due to apoptotic cell death. We chose two cell lines that were sensitive to MK-2206, MM1S and OPM2 and one cell line that was resistant to MK-2206, U266 to examine if % apoptotic cells increased post drug treatment. For this, we incubated MM1S, OPM2 and U266 cells with indicated concentrations of MK-2206 for 48 hrs and examined for apoptotic cells by annexin/PI staining. In both the sensitive cell lines examined, MK-2206 induced a dose-dependent increase in cells undergoing apoptosis **(** **Figures 2A** **)**. We then incubated MM1S and OPM2 with 2.5 µM of MK-2206 for indicated time points and observed a time-dependent increase in apoptosis **(** **Figure 2B** **)**. However, MK-2206 was unable to induce apoptosis in the resistant cell line U266 **(Figure S1)**. The increase in apoptosis was mediated by both the intrinsic and extrinsic apoptotic pathways as shown by activation of caspases 9, 8 and 3 in both the sensitive cell lines MM1S and OPM2 **(** **Figures 2C, D** **)**. In U266, as expected, we did not observe activation of any of the caspases **(Figure S2)**. In order to further understand if caspase activation is essential for MK-2206 to induce apoptosis, we treated MM1S and OPM2 cells with 10 µM of the pan-caspase inhibitor Q-VD-OPH in combination with MK-2206. Q-VD-OPH was able to partially protect both MM1S and OPM2 cells from cell death induced by MK-2206 **(** **Figures 2E and F** **)**. This effect was more pronounced in OPM2 cells where IC50 increased from 1 µM to 5 µM when cells were cultured with MK-2206 in the presence of Q-VD-OPH. This indicated that though caspase activation was important for MK-2206 to induce cell death, caspase independent mechanisms could also be involved. To further examine whether the extrinsic or intrinsic pathways were more involved in the partial protection, we treated MM1S and OPM2 cells with 10 µM of Z-IETD-FMK (a caspase 8 specific inhibitor) or 10 µM of Ac-LEHD-CMK (a caspase 9 specific inhibitor) and MK-2206. Treatment with either of these inhibitors was not able to protect MM cells from MK-2206 induced cell death indicating that both the extrinsic and intrinsic pathways were activated by the drug **(** **Figures 2E and F** **)**.

Next, we examined if MK-2206 was able to induce apoptosis in primary cells from MM patients. As shown in **figures 2G** **,** MK-2206 was able to induce apoptosis in primary cells from MM patients. However, like in MM cell lines, few MM patient cells were sensitive and few were resistant to MK-2206 treatment in vitro. MK-2206 did not induce apoptosis in lymphocyte populations indicating the specificity of MK-2206 to MM cells **(data not shown)**. We have however not performed any studies examining the effect of MK-2206 on other hematopoietic subsets which is a limitation of this study. We also examined the cytogenetic abnormalities in these patients based on FISH studies done as part of their clinical care, in order to identify any specific abnormalities that might predict for sensitivity to MK-2206. We did not observe any particular cytogenetic abnormality that predicted for sensitivity to the drug **(data not shown)**.

### MK-2206 Inhibits Activated Akt and Downstream Members of the Akt Pathway

**Figure 3:**
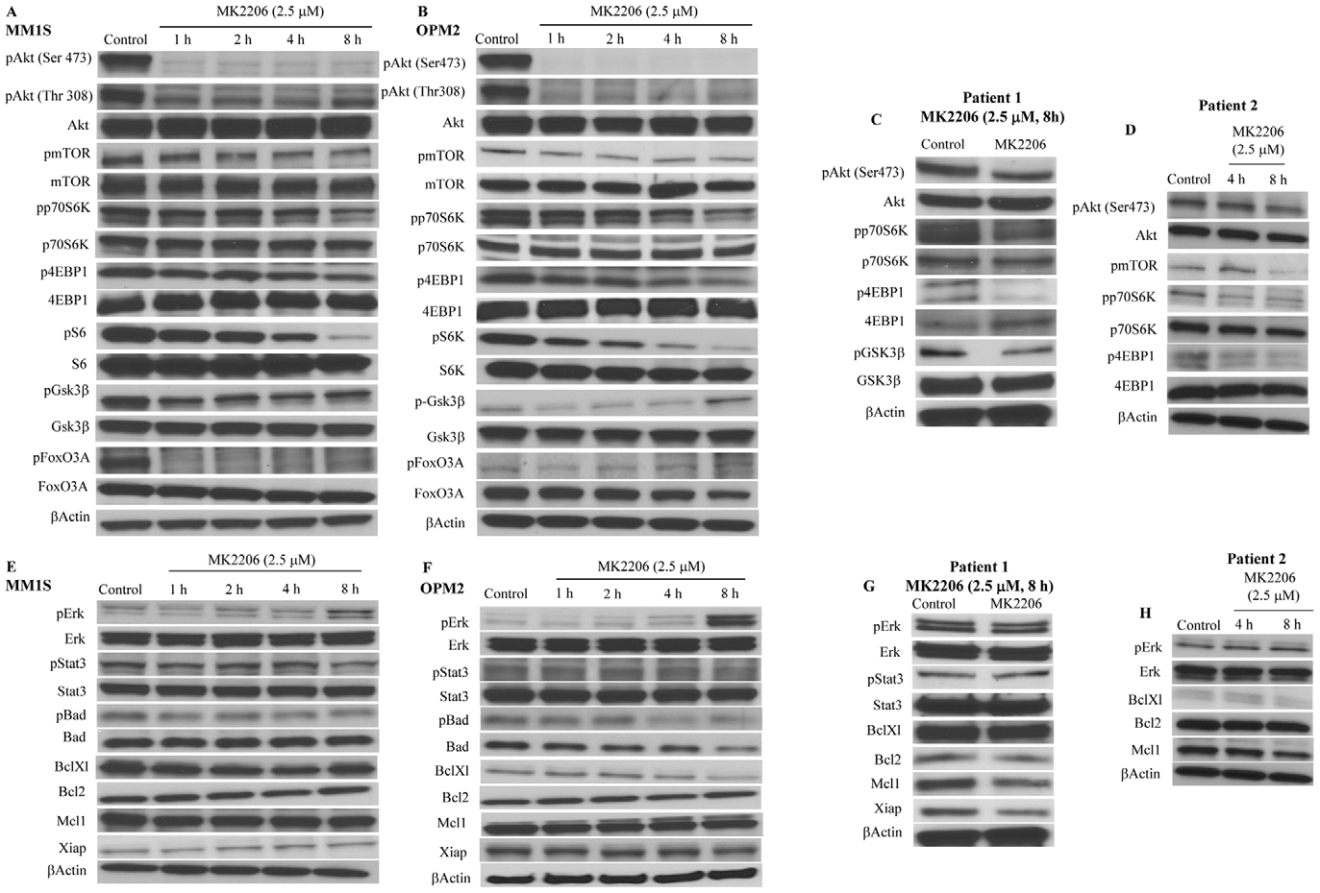
MK-2206 inhibits pAkt and downstream members of the PI3K/Akt/mTOR pathway and modulates expression levels of other signaling pathway proteins. We incubated **A and E)** MM1S or **B and F)** OPM2 cells with 2.5 µM of MK-2206 for 1, 2, 4 or 8 hrs. We observed down regulation of pAkt and downstream members of the pathway in both MM1S and OPM2 cells. We observed up regulation of pErk in both cell lines. We also observed down regulation of pBad and Bcl-Xl. No differences were observed in total proteins and actin, which serve as loading controls. We incubated CD138+ cells from **C and G)** Patient 1 or **D and H)** Patient 2 with 2.5 µM of MK-2206 for indicated time points and observed down regulation of pAkt, pmTOR, p-p70S6K, p4EBP1 and pGsk3β. We observed down regulation of Mcl1 and Xiap levels in patient 1 and down regulation of Mcl1 in patient 2. In all the above experiments control refers to cells untreated with MK-2206.

From **figure 1F** it was clear that MK-2206 was able to substantially inhibit pAkt after 8 hrs of incubation with the drug and this decrease was observed even in the presence of IGF. However, pmTOR levels were not inhibited to similar extent in the presence or absence of IGF. We therefore examined the effects of MK-2206 in more detail on both PI3K/Akt/mTOR pathway and other signaling pathway proteins that cross talk with this pathway. For this, we used MM1S and OPM2 cells and incubated them with 2.5 µM of MK-2206 for indicated time points. MK-2206 was able to inhibit pAkt (both Ser 473 and Thr 308) levels in both cell lines after as early as 1 hr of treatment. Activated levels of proteins down-stream of Akt including mTOR, p70S6K and 4EBP1were also down regulated though not to a similar degree **(** **Figures 3A, B** **)**. Other substrates of Akt including pS6 and pFoxO3A were significantly down regulated in MM1S and OPM2 cells **(** **Figures 3A and B** **)**. However, in OPM2 cells pGsk3β showed only a transient down regulation after MK-2206 treatment with higher levels of expression observed after 8 hrs of drug treatment **(** **Figure 3B** **)**. Thus, MK-2206 greatly inhibited Akt activity as observed by down regulation of its substrates. We then examined if MK-2206 was able to inhibit pAkt in patient cells extracted from myeloma patients. For this, we purified CD138+ plasma cells from two myeloma patients and incubated them with indicated concentration of MK-2206 for indicated time points. Like in MM cell lines, MK-2206 was able to inhibit pAkt in both patient cells and the inhibition of Akt activity translated to lower levels of pmTOR, p-p70S6K, p4EBP1 and pGsk3β **(** **Figures 3C, D** **)**. It must be noted that due to limited sample availability from the two patients, we were unable to monitor the expression levels of other pAkt substrates in the patient cells.

### Additional Mechanisms are Involved in the Anti-myeloma Activity of MK-2206

In MM, the PI3K/Akt/mTOR pathway crosstalks with other important signaling pathways implicated in myeloma including the MEK/Erk and Jak/Stat pathways [33], [34]. Hence, we wanted to examine if MK-2206 treatment modulates the expression levels of the above mentioned pathway proteins. As shown in **figures 3E and F**, MK-2206 was not observed to modulate pStat3 levels whereas pErk levels were up regulated at the concentration used in both MM1S and OPM2 cells respectively. However, in the primary cells from two MM patient cells (the same patients as above), MK-2206 treatment did not lead to upregulation of pErk **(** **Figures 3G, H** **)**.

Next, we wanted to examine the effect of MK-2206 on the pro and anti-apoptotic proteins. Bad, a pro apoptotic BH3 only protein of the Bcl2 family is a direct substrate of Akt and is phosphorylated by activated Akt preventing its binding to Bcl-Xl. MK-2206 down regulated pBad levels in both MM1S and OPM2 cells. Upon examining levels of anti-apoptotic proteins post MK-2206 treatment, we observed no difference in the expression levels of Bcl2, Mcl1 and Xiap and down regulation of Bcl-Xl in both cell lines tested **(** **Figures 3E, F** **)**. In the two patient cells, however, MK-2206 treatment caused a significant down regulation of Mcl1 and no significant impact on Bcl2 and Bcl-Xl levels **(** **Figures 3G, H** **)**. Thus, MK-2206 treatment could induce apoptosis through different mechanisms down-stream of Akt in different cells mainly due to the wide array of proteins modulated by activated Akt.

### Inhibition of PI3K/Akt Pathway at Multiple Levels Result in Synergistic Anti-myeloma Activity

**Figure 4:**
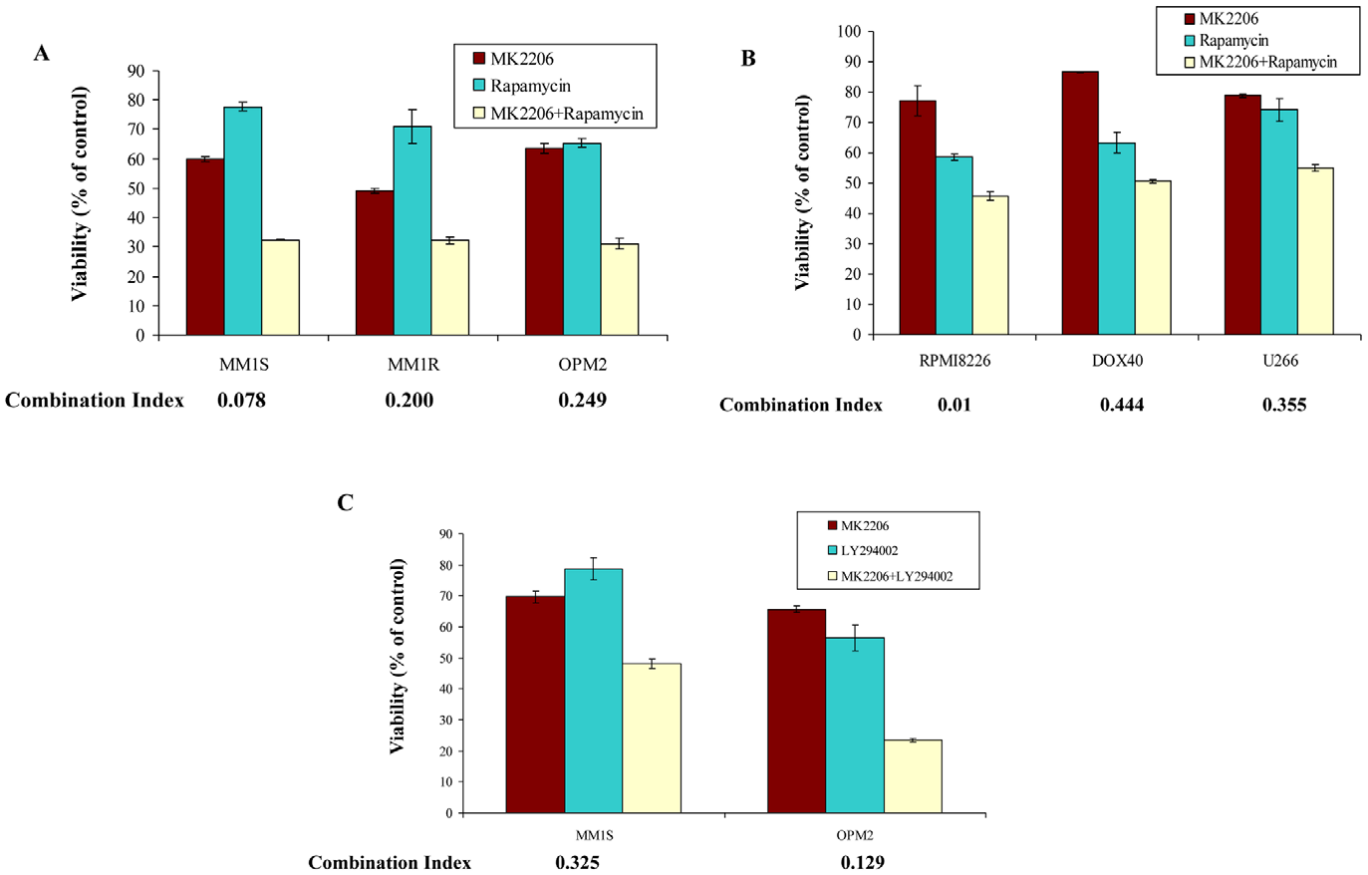
MK-2206 synergizes with other inhibitors of the PI3K/Akt/mTOR pathway. When MK-2206 was combined with the mTOR inhibitor rapamycin, synergistic cytotoxicity was observed **A)** in cell lines sensitive to MK-2206 (MM1S, MM1R and OPM2) and **B)** in cell lines less sensitive to MK-2206 (RPMI8226, DOX40 and U266) as a single agent as measured using the MTT assay. The graph represents the concentrations of MK-2206, rapamycin or the combination at which maximum synergy was observed. The concentrations are: 1) MM1S−0.5 µM MK-2206, 5 nM rapamycin, 2) MM1R−0.5 µM MK-2206, 5 nM rapamycin and 3) OPM2−0.5 µM MK-2206, 5 nM rapamycin. 4) RPMI8226−2.5 µM MK-2206, 20 nM rapamycin, 5) DOX40−2.5 µM MK-2206, 5 nM rapamycin and 6) U266−5 µM MK-2206, 10 nM rapamycin. Cell lines used are indicated on the X-axis and Viability (% of control) indicated on the Y-axis. Error bars represent standard deviation. **C)** When MK-2206 was combined with the PI3K inhibitor LY294002, synergistic cytotoxicity was observed in cell lines sensitive to MK-2206 (MM1S and OPM2) as a single agent as measured using the MTT assay. The graph represents the concentrations of MK-2206, LY294002 or the combination at which maximum synergy was observed. The concentrations are: 1) MM1S−0.5 µM MK-2206, 10 µM LY294002 and 2) OPM2−0.5 µM MK-2206, 10 µM LY294002. Cell lines used are indicated on the X-axis and Viability (% of control) indicated on the Y-axis. Error bars represent standard deviation.

From the results so far, it is clear that lack of dependence on the Akt in some cell lines precludes a relevant level of cytotoxicity with Akt inhibitors. Hence, we wanted to examine if simultaneously inhibiting the pathway at multiple levels or inhibiting multiple pathways can result in synergistic effects. We first incubated three sensitive cell lines MM1S, MM1R and OPM2 with various concentrations of MK-2206 and rapamycin for 48 hrs**.** In all the three cell lines, we observed marked synergy. The results presented represents the concentration at which maximum synergy was observed **(** **Figure 4A** **)**. It is well known that mTOR inhibition leads to activation of Akt. Therefore, we wanted to test if using rapamycin with MK-2206 could sensitize the MM cell lines that do not express basal levels of activated Akt and were less sensitive to MK-2206 treatment. MK-2206 was observed to synergize with these cell lines as well at various concentrations. The results presented in **figure 4B** represents the concentration at which maximum synergy was observed in RPMI8226, DOX40 and U266 cells. It is also known that p-p70S6K when inhibited leads to up regulation of the PI3K/Akt pathway by activating insulin receptor substrate (IRS), which might also contribute to resistance in cells exposed to MK-2206 treatment. We therefore wanted to examine MK-2206 in combination with the PI3K inhibitor LY294002. Like in combination with rapamycin, MK-2206 synergized with LY294002 in killing cell lines sensitive to MK-2206 treatment **(** **Figure 4C** **)**. However, we did not observe synergy when MK-2206 was used in combination with LY294002 in MM cell lines less sensitive to MK-2206, as expected since Akt status probably does not play a role in the survival of these cells. **(data not shown)**.

### Activation of MEK/Erk Pathway Plays an Important Role in Resistance to Akt Inhibition in Myeloma Cells

**Figure 5:**
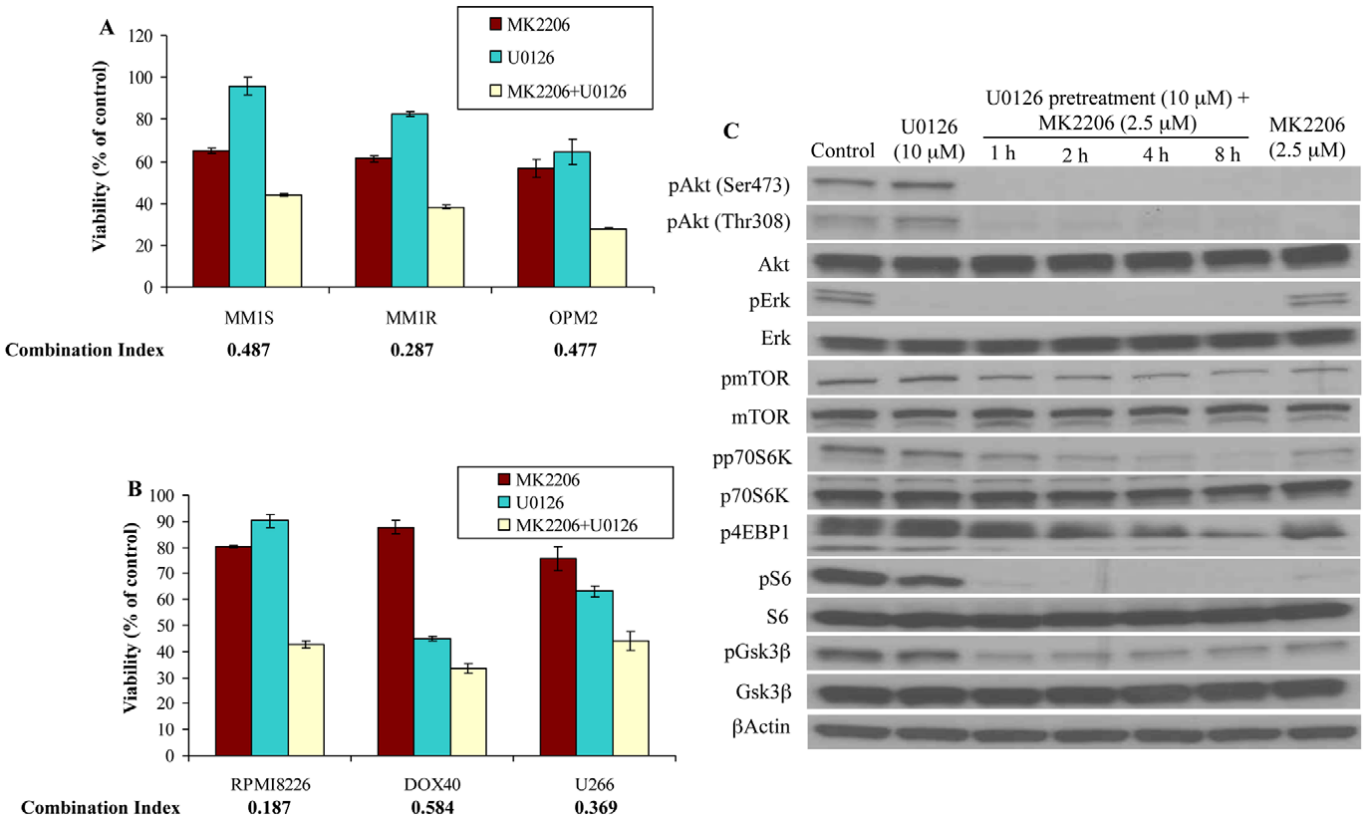
MK-2206 synergizes with the Mek inhibitor U0126 and the drug combination leads to increased inhibition of mTOR activity. When MK-2206 was combined with the Mek1/2 inhibitor U0126, synergistic cytotoxicity was observed **A)** in cell lines sensitive to MK-2206 (MM1S, MM1R and OPM2) and **B)** in cell lines less sensitive to MK-2206 (RPMI8226, DOX40 and U266) as a single agent as measured using the MTT assay. The graph represents the concentrations of MK-2206, U0126 or the combination at which maximum synergy was observed. The concentrations are: 1) MM1S−0.5 µM MK-2206, 10 µM U0126, 2) MM1R−0.5 µM MK-2206, 10 µM U0126 and 3) OPM2−1 µM MK-2206, 20 µM U0126. 4) RPMI8226−2.5 µM MK-2206, 10 µM U0126, 5) DOX40−5 µM MK-2206, 20 µM U0126 and 6) U266−5 µM MK-2206, 30 µM U0126. Cell lines used are indicated on the X-axis and Viability (% of control) indicated on the Y-axis. Error bars represent standard deviation. **C)** MM1S cells were left untreated or treated with U0126 (10 µM) for 24 hrs. Following this, cells were treated with 2.5 µM of MK-2206 for 1, 2, 4 or 8 hrs. As a control, MM1S cells were treated with U0126 alone (10 µM) for 32 hrs or MK-2206 alone (2.5 µM) for 8 hrs. We observed significant down regulation of pAkt and other downstream members of the pathway and pErk when MK-2206 was used in combination with U0126 with no difference in total proteins and actin, which serve as loading controls.

Constitutive levels of activated Erk are observed in almost all MM cell lines examined with increased levels observed in cell lines lacking pAkt expression and less sensitive to MK-2206 **(** **Figure 1C** **)**. Also, MK-2206 treatment led to an upregulation of pErk in the two MM cell lines used **(** **Figures 3E and F** **)**. In addition, we observed that MK-2206 potently inhibited pAkt levels without comparable level of inhibition of the proteins down-stream of pAkt **(** **Figures 3A and B** **)**. It is known that activated Erk feeds into the PI3K/Akt/mTOR pathway at the mTOR level and hence we hypothesized that this could be a factor contributing to the lack of significant pmTOR inhibition [23]. We therefore examined if MK-2206 can synergize with inhibitors of the Mek/Erk pathway. More importantly, we examined whether pretreating the cells less sensitive to MK-2206 with a MEK inhibitor would sensitize these cells to MK-2206 through mTORC2 induced pAkt activation. To test this, we pretreated MM cells with indicated concentrations of U0126 for 24 hrs. Following this, we treated these cells with indicated concentrations of MK-2206 for 48 hrs and examined the viability of cells. We observed marked synergy when MK-2206 was used in combination with U0126 both in cell lines sensitive to MK-2206 **(** **Figure 5A** **)** and more importantly in lines less sensitive to MK-2206 including RPMI8226, DOX and U266 **(** **Figure 5B** **)**. We then examined the mechanism of action of the combination to better understand the synergistic response observed. For this, we pretreated MM1S cells with 10 µM of U0126 for 24 hrs. Following this, we treated these cells with 2.5 µM of MK-2206 for 1, 2, 4 or 8 hrs. As controls, we treated MM1S cells with just U0126 or MK-2206 or left them untreated. We then examined the levels of pAkt, pErk, pmTOR, p-p70S6K, p4EBP1, pS6 and pGsk3β. We observed that MK-2206 clearly inhibited pAkt alone or in combination with U0126 **(** **Figure 5C** **)**. U0126, as expected, leads to up regulation of pAkt (both Ser 473 and Thr308) which was efficiently down-regulated by MK-2206 **(** **Figure 5C** **)**. U0126 clearly inhibited pErk to similar extents as a single agent or in combination with MK-2206 **(** **Figure 5C** **)**. The combination of MK-2206 and U0126 inhibited mTOR activity more effectively than either of these agents as single agents **(** **Figure 5C** **)**. Similarly, pGsk3β that is down-stream of both pAkt and pErk, was down-regulated more significantly when MM1S cells were treated with the drug combination **(** **Figure 5C** **)**. This once again indicated that activated Erk feeds into the PI3K/Akt/mTOR pathway and inhibiting pErk could sensitize cells to PI3K pathway inhibitors.

**Figure 6:**
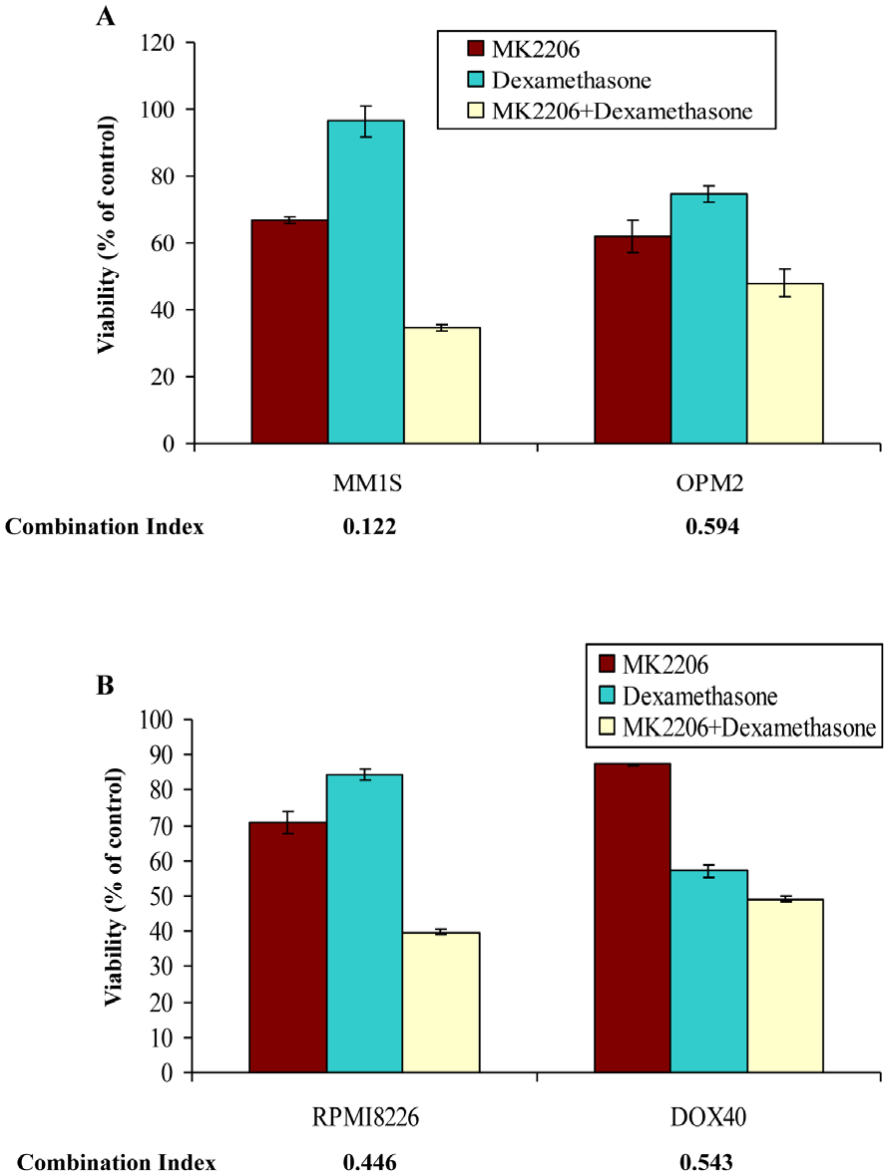
MK-2206 synergizes with dexamethasone. When MK-2206 was combined with dexamethasone, synergistic cytotoxicity was observed **A)** in cell lines sensitive to MK-2206 (MM1S and OPM2) and **B)** in cell lines less sensitive to MK-2206 (RPMI8226 and DOX40) as a single agent as measured using the MTT assay. The graph represents the concentrations of MK-2206, dexamethasone or the combination at which maximum synergy was observed. The concentrations are: 1) MM1S−0.5 µM MK-2206, 10 nM dexamethasone and 2) OPM2−1 µM MK-2206, 40 nM dexamethasone. 3) RPMI8226−5 µM MK-2206, 20 nM dexamethasone and 4) DOX40−2.5 µM MK-2206, 50 nM dexamethasone. Cell lines used are indicated on the X-axis and Viability (% of control) indicated on the Y-axis. Error bars represent standard deviation.

Finally, given the significant role of dexamethasone as a therapeutic agent for myeloma we examined if pAkt inhibition can synergize with corticosteroid therapy. The PI3K/Akt/mTOR pathway is also known to be a critical pathway contributing to dexamethasone resistance in MM cells [20], [37]. For example, activated PI3K/Akt pathway inactivates caspase-9 and offers resistance against Dexamethasone induced apoptosis [20]. We therefore examined the cytotoxicity of the combination of MK-2206 and dexamethasone in MM cell lines. We observed marked synergy when the drugs were used in combination in both cell lines sensitive to MK-2206 **(** **Figure 6A** **)** and those resistant to MK-2206 **(** **Figure 6B** **)**.

## Discussion

The PI3K/Akt/mTOR pathway has long been shown to be important in MM disease progression and resistance to therapy. However, the limited availability of drugs specifically targeting this pathway has been a limiting factor to understand the clinical benefits of inhibiting this pathway. In addition, mTORC1 inhibitor rapamycin treatment resulted in a cytostatic effect with minimal cytotoxicity in various tumor systems [38]. The reasons for this are now better understood and have been attributed mainly to increased Akt activation by mTORC2 [26]. Over the last few years numerous drugs specifically targeting this pathway including PI3K, Akt and pan-mTOR inhibitors have been developed. In MM, recent reports present promising preclinical data on pan-mTOR inhibitors and dual inhibitors of PI3K and mTOR [39], [40], [41], [42], [43]. Perifosine, an alkylphospholipid has been found to inhibit proliferation and induce apoptosis in MM cells by inhibiting Akt activity [36], [44]. A clinical trial using perifosine in combination with bortezomib in bortezomib refractory or bortezomib and dexamethasone refractory relapsed myeloma patients are encouraging with an overall response rate of 40% [45]. However, it must be noted that perifosine in addition to Akt inhibition could also modulate other signaling pathways [36], [46] and anti-MM effects of using a specific Akt inhibitor have not been evaluated. We therefore wanted to examine an Akt kinase specific inhibitor both for pre-clinical validation and also to better understand the PI3K/Akt/mTOR pathway in MM cells.

MM patients could present with various cytogenetic abnormalities including t(4;14), t(11;14), t(14;16), del(17p), abnormalities of chromosomes 1 and 13 and trisomies of various other odd numbered chromosomes. Some abnormalities, especially deletions and monosomies involving chromosomes 1, 13 and 17 can be acquired with disease progression. We therefore wanted to examine the cytotoxic effects induced by MK-2206 in a panel of MM cell lines representative of the various abnormalities observed in MM patients. MK-2206 was found to be extremely sensitive in killing MM cells with t(4;14) translocations (H929, OPM2) irrespective of their p53 status (del17p) since H929 expresses wt p53 whereas OPM2 has mutated p53. MM1S and MM1R, cell lines with t(14;16) translocation were sensitive to MK-2206 treatment whereas RPMI8226, DOX40 and LR5 with the same translocation were less sensitive to MK-2206 treatment. Thus, none of the translocations appear to predict for sensitivity to MK-2206. We therefore examined the baseline constitutively activated levels of pAkt and down-stream members of the Akt pathway and observed a positive correlation between levels of pAkt and sensitivity to the drug**.** We demonstrated potent inhibition of proliferation of myeloma cells by MK-2206 even when co cultured with BMSCs and cytokines. We present data indicating that this effect is due to the specific and potent inhibition of pAkt (both Thr 308 and Ser 473) and down-stream members of the Akt pathway in both MM cell lines and patient cells. Clearly, both mTORC1 and mTORC2 activity is inhibited as shown by reduction in levels of p-p70S6K, p4EBP1 (mTORC1 substrates) and pAkt (Ser 473) (mTORC2 substrate). Though we observed inhibition of pmTOR and its substrates, the extent of the down regulation was not as pronounced as pAkt down regulation. It has been reported that p-p70S6K inhibition leads to PI3K mediated up regulation of the Mek/Erk pathway [47]. Additionally, activated Erk has been shown to phosphorylate TSC2 and raptor, both events leading to activation of mTOR [38], [48]. It has long been suggested that the Mek/Erk pathway and the PI3K/Akt pathway cross inhibit each other and this cross inhibition is why inhibitors of either pathway lead to upregulation of the other pathway [49], [50]. In our studies, we observed that MK-2206 either had no effect on pErk levels or in some cases increased pErk levels. In OPM2 cells where we observed significant up regulation of pErk post MK-2206, we also observed the lack of down regulation of pGsk3β. Gsk3β has been shown to be phosphorylated by activated Erk in addition to pAkt and this once again indicated the complex interaction between these two pathways [51]. Pre treating MM cells with U0126 (Mek1/2 inhibitor) followed by MK-2206 treatment demonstrated synergy in killing MM cells and our results clearly indicated that the drug combination inhibited mTOR activity significantly. In addition, it has recently been shown that Akt inhibition leads to reactivation of multiple receptor tyrosine kinases in several different tumor systems through the inhibition of Akt induced negative feedback loop [52]. Thus it is very likely that additional mechanisms could contribute to resistance to MK-2206 in MM cells. We are currently examining the activated levels of Akt and Erk in MM patient cells to evaluate if these could serve as biomarkers to predict for sensitivity to MK-2206 and other PI3K/Akt/mTOR inhibitors.

Our results clearly indicate the complex nature of the PI3K/Akt/mTOR pathway, the benefits of inhibiting this pathway at multiple levels and the synergistic effect of simultaneously inhibiting both this and the Mek/Erk pathways in MM cells. Specifically, we present evidence in support of the Mek/Erk pathway driving the down-stream elements of the Akt pathway, providing a resistance mechanism in myeloma cells. Taken together, we present clear evidence for MK-2206 alone or in combination with rapamycin, a PI3K inhibitor, an inhibitor of the Mek/Erk pathway or dexamethasone to be taken up for clinical investigation in MM patients, and most importantly, to use baseline expression of pAkt and pERK as companion biomarkers in patient selection for clinical trials with this agent.

## Supporting Information

Figure S1 Apoptosis is not induced by MK2206 in U266 cell line. We incubated U266 cells with **A)** indicated concentrations of MK-2206 for 48 hrs and **B)** with 2.5 µM of MK-2206 for 12, 24 or 48 hrs. We observed absence of dose and time dependent increase in apoptosis as measured by annexin/PI staining. % cells in each quadrant is indicated. In all the above experiments, control refers to cells untreated with MK-2206.
(TIF)

Figure S2 Caspases are not activated by MK2206 in U266 cell line. U266 cells were incubated with indicated concentrations of MK-2206 for 48 hrs and we measured activation of caspases 9, 8 and 3. None of the caspases were activated by MK2206. In all the above experiments, control refers to cells untreated with MK-2206.
(TIF)

Table S1 IC50 values of MK2206 on MM cell lines. This table indicates the IC50 values of MK2206 on all MM cell lines examined. From this table, it is clear that MK2206 exhibits preferential cytotoxicity on a few MM cell lines.
(PDF)
