# Supplementary figures and images for: Anti-Myeloma Activity of Akt Inhibition Is Linked to the Activation Status of PI3K/Akt and MEK/ERK Pathway
Source: PLoS One. 2012 Nov 21;7(11):e50005. doi: 10.1371/journal.pone.0050005 (PMC3503708; doi:10.1371/journal.pone.0050005)

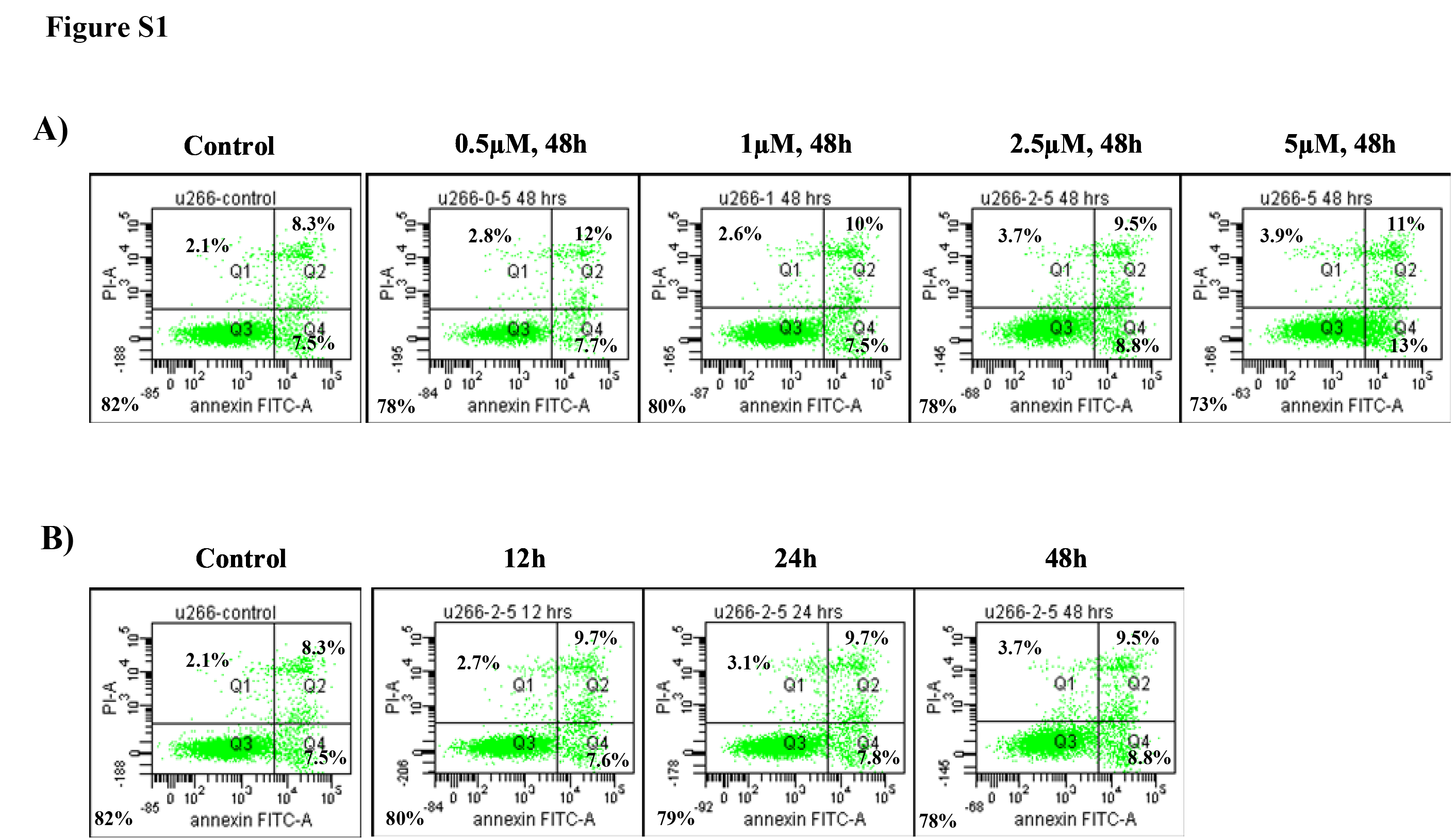

Supplement: Figure S1 — Apoptosis is not induced by MK2206 in U266 cell line. We incubated U266 cells with A) indicated concentrations of MK-2206 for 48 hrs and B) with 2.5 µM of MK-2206 for 12, 24 or 48 hrs. We observed absence of dose and time dependent increase in apoptosis as measured by annexin/PI staining. % cells in each quadrant is indicated. In all the above experiments, control refers to cells untreated with MK-2206. (TIF) [file pone.0050005.s001.tif]

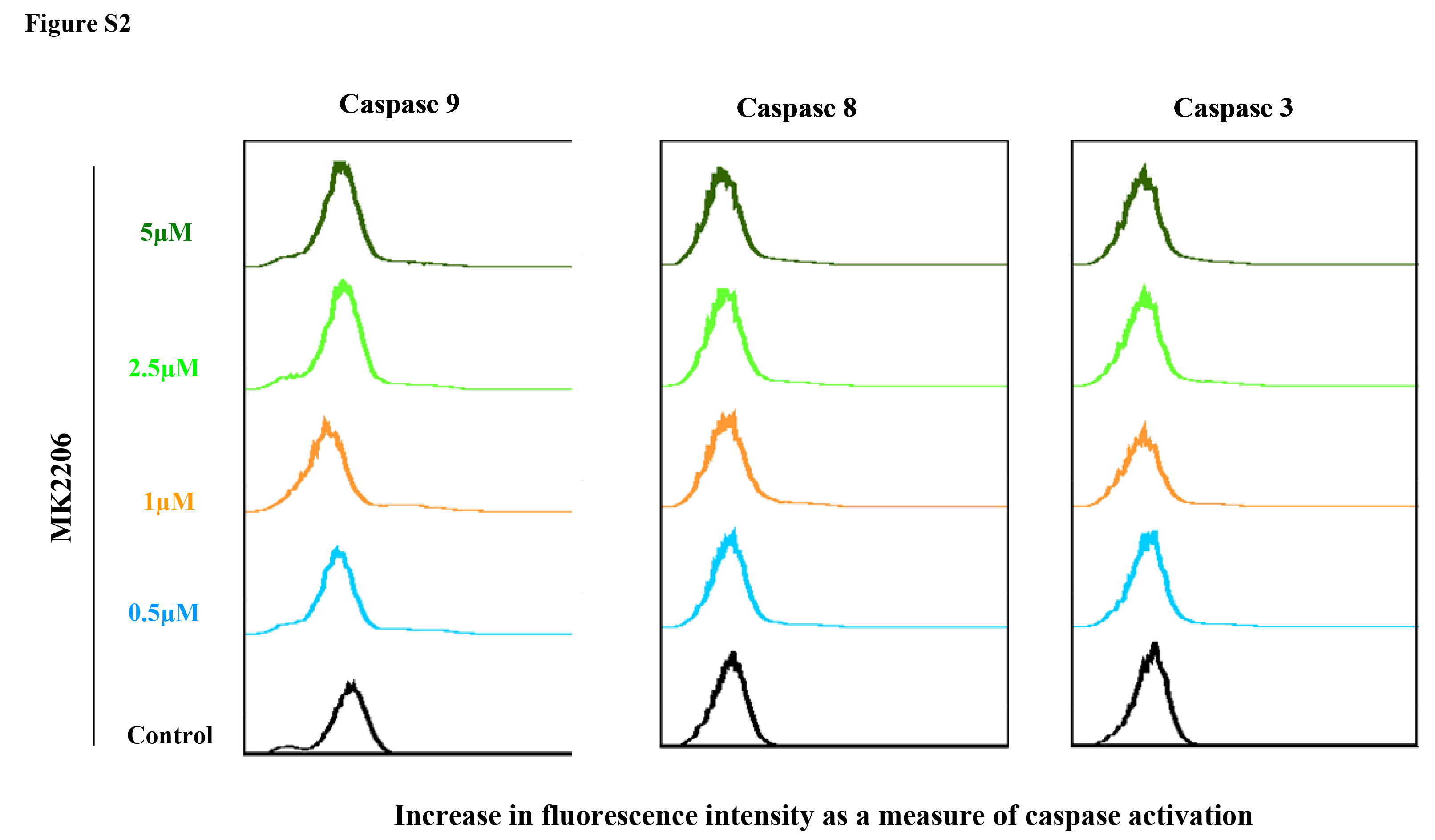

Supplement: Figure S2 — Caspases are not activated by MK2206 in U266 cell line. U266 cells were incubated with indicated concentrations of MK-2206 for 48 hrs and we measured activation of caspases 9, 8 and 3. None of the caspases were activated by MK2206. In all the above experiments, control refers to cells untreated with MK-2206. (TIF) [file pone.0050005.s002.tif]
