# Supplementary material for: Anti-Myeloma Activity of Akt Inhibition Is Linked to the Activation Status of PI3K/Akt and MEK/ERK Pathway
Source: PLoS One. 2012 Nov 21;7(11):e50005. doi: 10.1371/journal.pone.0050005 (PMC3503708; doi:10.1371/journal.pone.0050005)
Supplement: Table S1 — IC50 values of MK2206 on MM cell lines. This table indicates the IC50 values of MK2206 on all MM cell lines examined. From this table, it is clear that MK2206 exhibits preferential cytotoxicity on a few MM cell lines. (PDF) [file pone.0050005.s003.pdf]

Table S1

Cell Line	***IC_{50} (μM)***
MM1S	1.0
MM1R	0.5
OPM2	2.5
H929	0.5
RPMI8226	10.0
DOX40	15.0
LR5	Not Reached
U266	10.0
